# Eliciting Challenges on Social Connectedness among Filipino Nurse Returnees: A Cross-Sectional Mixed-Method Research

**DOI:** 10.1155/2016/9187536

**Published:** 2016-08-02

**Authors:** Mary Jane L. Cortez, Christian V. Del Rosario, Michael Joseph S. Diño

**Affiliations:** ^1^The Graduate School, University of Santo Tomas, 1015 Manila, Philippines; ^2^Research Development and Innovation Center, Our Lady of Fatima University, 1440 Valenzuela, Philippines

## Abstract

This cross-sectional study utilized a nested concurrent design to determine the association of Filipino nurse returnees' length of stay since they returned and their social connectedness as well as the essence of communication from their perspective. The respondents, who are Filipino nurses (*n* = 107) who worked abroad and returned to the Philippines for good, were employed from June to July of 2015 via referrals from colleges and institutions in Metro Manila and Bulacan areas in the Philippines. The quantitative results revealed, in one hand, significant but weak correlation between the respondent's length of stay and social connectedness (*r* = 0.224, *p* = 0.021, *α* = 0.05). On the other hand, three themes were generated from the qualitative analysis, namely, (1) Taking-In, (2) Taking-Hold, and (3) Letting-Go. The Social Connection System (SCS) provides a visual depiction of the social connectedness of a person. This research is geared towards the understanding of the interesting phenomenon of migration and social coherence of Filipino professionals.

## 1. Introduction

Return of migrants to their homeland is a phenomenon of many faces and phases, and it is absolutely true that this life event is very interesting to research on. Return is defined as the event when a migrant goes back to his or her homeland after being in a country for a certain period of time [1]. For many, returning home is perceived with cherished getting together and making up for lost times [2, 3], but returns are also afflicted with numerous unfavorable experiences, such as indifference, social discomfort, and painful adjustments [2, 4].

Currently in the Philippines, there is prevailing culture of migration among Filipino nurses, that is, Filipino nursing professionals going abroad to seek employment [5]. But despite this demand for nurses outside the country, there is also an emerging trend of increasing number of Filipino nursing migrants returning home for good. Four million Overseas Filipino Workers (OFWs) were projected to have permanently returned to the Philippines [6]; but this includes all OFWs with variety of professions and expertise. This makes it even more interesting to look on the experiences of Filipino nurse professionals who went home permanently.

Using a mixed-method research, this cross-sectional study explored on the social experiences of nurses who returned to the Philippines, while being supported by numerical data. The qualitative phase centered its inquiry on defining the essence of communication among Filipino nurse migrants upon their return to the country. Quantitative phase tested on the relationship between length of stay after return and social connectedness of the participants. While the term return may have variations in its definition, such as returning for good, or returning for a temporary period of time, this study limited its focus to Filipino nurse migrants who returned to the Philippines permanently.

The study is designed for readers to understand the robust phenomenon of nursing professionals who sought good life in foreign countries and returned back to their native land, using radical research data gathering techniques.

## 2. Background

### 2.1. Theoretical Framework

The stress and coping approach to change was derived from various psychological models on impacts of live events [7]. This approach stemmed from the conjecture that changes in life events are stressful in nature and that a person who undergoes these changes is subjected to a process of shock and adaptation. Shock or stress is the initial indications of stressful life changes, so a person needs to develop coping strategies. Developing these strategies indicates the adaptation phase of this process.

Interestingly, the process of adaptation is influenced by a number of variables including the person's preparation, the degree of change, personality factors, and social support. These factors determine the convenience of the process [7].

Nursing professionals who go back to the Philippines for good experience life changes in a similar fashion. Their return is accompanied by emotional stresses, and they are expected to undertake adaptation process. This study, however, would focus only on the social connectedness of the nursing professionals. The study revolved around this framework.

### 2.2. Variable Discussion

#### 2.2.1. Migration

Migration is basically defined as a temporary or permanent displacement of a person from its usual residence or home country to another in a set period of time [8].

There are many theories, individual and structural, that centered its focus on migration of people. These theories mostly explicate the determinants of migration to other countries. One factor is economic disparities, such as wage differentials, employment opportunities, and inequalities in the standards of living; that is, when the home country commonly provides little opportunities or the person feels unsatisfied with his or her current standard of living, he or she will most likely migrate to another country with desirable economic environment. Another factor is security in terms of human and environment. Social disparities involve an individual's cultural characteristics which may either connect or disconnect with one's country and opportunities for education [9].

Migration is plagued with negative experiences from migrants. Initially, migrants experience confusion on the how-to's of the country, like riding trains. Migrants also experience social and cultural disconnection from locals or migrants from other countries. Some even report being abused by employers. Emotional problems such as homesickness and longing for family are perceived as one hard difficulty to recover on [10, 11].

Nonetheless, migration is also connected to many perceived positive effects, such as a better standard of living, access to good education, enhanced support from public and government institutions, and opportunities for understanding and adapting to different cultural practices. There is also, at a certain degree, intellectual gain from migrants, when there were detected improvement in their qualifications and acquisition of critical skills and knowledge on new technologies while working abroad [12, 13].

#### 2.2.2. Return Migration

A person's return to his or her homeland may seem the end of his or her journey, but theorists and researchers no longer regard it as the last step of the process, since returnees undergo even more stages in life once they return to their country (Vlase, 2011 cited in [13]). This involves the stresses and coping of migrant returnees as formerly discussed in this paper.

A voluminous number of migrants are very explicit of their reasons for return, which gears towards family reasons. This is not unusual since researches found family to be a primary determinant of migrants' reason for return. It can be in a form of desire to be near parents or, if they are the parent, children. It may sometimes be triggered by death or illness of a family member, simply to be able to devote some more time while they are still alive [14, 15]. Parents who have dependents at home have higher chance of returning [14].

The migrants' duration of stay and income are also a contributing factor for returning. Professionals who have been staying in the host country for longer time are much more likely to return. Likewise, the odds ratio for returning decreases with higher income. But it is remarkable to note though that, among professionals, healthcare providers have the highest probability to return to its home country [14].

While there has been increasing attention in the issue of migration in through different research studies [16], it is interesting to know that there is so much little knowledge or studies conducted about return migrants [13, 17] particularly in the Philippines. It is observed by researchers that perhaps most researches on return migration are conducted on highly developed countries, and therefore little emphasis was given on the return migrants who actually came from the developing ones [13]. This is unfortunate because there is supposed value on understanding the perceptions about return migration from the lens of the immigrants themselves and their home communities, their homeland. The former author even mentioned the term “remigration” as considered by a group of researchers the final stage in this process [13].

This creates an emergence to conduct qualitative and quantitative studies focusing on Filipino nurse returnees.

#### 2.2.3. Social Connectedness

Social connectedness is defined as the relationship of an individual with the society [2]. It is pictured as a schematic pattern found in interpersonal relationships that is crucial for the psychological adjustment of a person [18–20]. In a deeper sense, social connectedness is a person's desire to form and maintain social bonds, to feel a sense of “relatedness” [21] and to identify himself or herself with the people they interrelate with [20]. It is a more pervasive process of feelings, thoughts, and behaviors in social situations [22].

Returning to one's homeland is not exactly a smooth ride, as one must undergo reintegration to the social environment of the homeland [13]. A positive adaptation to this stressful life event returns a positive social connectedness, that is, a friendly and approachable attitude and sense of meaning. On the other hand, a person who had not adapted well feel isolated, lonely, anxious, and depressed [2, 4, 23, 24]. And sometimes, it is even accompanied by health risks [23, 24].

Indeed, social connectedness can be thought of as an output of a person's way of coping, as discussed previously as the study framework. And returning to a person's country of origin is undoubtedly a stressful life experience but, if done well, could be a fulfilling learning process as well [4].

#### 2.2.4. Returnee Experiences

Return of migrants is a highly complex phenomenon. Little attention has been given on how returnees have reintegrated socially. Accordingly, among Filipino returnees, the social issues that became apparent were on dread of isolation and attainment of family goals. In fact, many Filipino returnees shared their intention of remigrating in the future [25]. Meanwhile, among return migrants from Netherlands to northeast Morocco, their sharing revealed the difficulty of reintegration locally in spite of the transnational efforts over the years. To a certain extent, returnees felt that upon coming back relatives show feelings of dislike and frustration towards them. The distance of migration has taken a toll on “relationships” over the years. A few cited the absence of the returnees during the time of illness and other caring activities shouldered by said relatives as a source of discomfort [26].

The remarkable changes in the character brought about by one's adaptation to the culture of a foreign land made them feel as “strangers in their own home” [27]. The females feel displaced compared to the male.

Women are more focused on the needs and happiness of their children. Abilities acquired abroad are not necessarily advantageous as a whole since on the downside, despite their improved qualifications and skills, returnees felt that they were not overtly received by the locals. The male returnees seemed to experience easier reintegration as compared to the female returnees. The males in the Caribbean appeared to have easily formed friendships and overcame feelings of being an “outcast,” while the females are warier in establishing union. The women were focused on the welfare of the children [26].

Sabates-Wheeler et al. [28] put emphasis on the need for prospects between the migrants and their families at home to be attuned. Vital to this will be the kind of information exchanged as to the realistic situation both in the home and host country and the openness to eventualities. In so doing, difficulties in the social environment can be mitigated. Migration cycle continues until the return of the migrant. Numbers pertaining to those who return must be considered just as important for those who are leaving.

On the other hand, return due to retirement does not necessarily mean that returnee can no longer make an impact on human capital of the home country. The question should be how a home country draws the attention of migrants in their prime to go back and share their abilities and experiences [29]. Moreover, people who feel they belong are more motivated. Social connectedness boosts the confidence of individuals to do more and do even better [24]. Individuals who have strong connection tend to be highly receptive to their influences. In fact, Lee et al. [30] discussed that as an individual grows into adulthood, he develops attachment to others whom he has similarities and an overall sense of self. Those who have high social connectedness easily establish relations. Meanwhile, individuals with low levels of connectedness consider themselves as misfits of society who have experienced constant disappointments. Further, low connectedness may result in inability to acquire interpersonal behaviors like being affable and intimate.

## 3. Research Method

### 3.1. Research Design

This is a cross-sectional study that utilized a mixed-method approach to data inquiry, specifically using nested concurrent design, wherein the samples employed in one phase is a subset of the samples in another phase (nested), with the data being gathered both at the same time (concurrent) [31].

For the quantitative phase of the study, descriptive correlational design is the most fitting to use due to its ability to describe behavior and measure the relationship between groups without changing its environment [32].

For the qualitative phase, the research interpretative phenomenology was used. The design intends to capture the Lebenswelt or life-world of a person (phenomenology) without the warrant of bracketing since prior understanding of the phenomenon is presumed before the conduct of the study (interpretative) [33]. This allows the researchers to engage with persons' lived experiences and explicitly enter into the process of data analysis through their preconceived reflections, or interpretations, of the participants' life-world [34]. This design is beneficial when the study warrants subjective perceptions from individuals while little information is known about its context (Smith & Osborn, 2007 cited in [35]).

### 3.2. Study Participants and Locale

The participants of the study were Filipino nurses who have worked abroad and are currently staying in the Philippines permanently and practicing their profession in an academic institution or a hospital. Due to the unavailability of official data on the number of returned nurse migrants over the years, a snowballing technique was utilized to gather more participants, wherein participants initially qualified to participate would refer other participants who might qualify to participate. Criteria for participant inclusion are as follows: (1) must be a Filipino nurse, (2) have worked their profession abroad, and (3) returned to the Philippines permanently to (4) practice their profession. All referred participants agreed to participate.

Participants selected for the study were interviewed for the qualitative phase. Considering the Lebenswelt principle of key informant selection [36], scholars believed that qualitative investigators (e.g., Heidegger) are recommended to select key informants with shared “universe” with the author [33]. It is then noted that all participants selected for the qualitative component were female and married, the same characteristic as of the interviewer assigned in the study. The researchers stopped interviewing participants as soon as data saturation was achieved at the 12th participant.

The study was conducted between June and July of 2015, covering various cities within Metro Manila and Bulacan area in the Philippines.

### 3.3. Instrumentation

The author utilized a 20-item questionnaire to measure the social connectedness of the respondents. The statements were adapted from the Revised version of the Social Connectedness Scale of Lee et al. [30]. To measure social connectedness, it asked various questions regarding the respondent's relationship with individuals, such as “I feel at ease in the presence of outsiders” and “I feel a sense of belonging to those around me.”

To ensure that the questionnaire is appropriate for target respondents, it underwent careful validation and was pilot tested with thirty (30) Filipino nurse returned migrants through a purposive sampling. Used in the measures was a 6-Point Likert Rating Scale (1 = strongly disagree to 6 = strongly agree). It yielded a Cronbach's Alpha score of 0.78 which suggests commendable reliability of the used questions.

A semistructured interview guide, or aide memoire, was created for the phenomenological component of the study. It consists of five questions with the central question of “What defines communication from the lens Filipino nurse migrants after returning to the country for good?”

### 3.4. Data Analysis

Mean scores were calculated to get the respondent's length of stay in the country since return as well as their social connectedness scores. Pearson Product-Moment Correlation was used to determine the direction and strength of association between the respondents' length of stay since return and social connectedness.

After the data gathering process, the data was tallied using a spreadsheet software for Windows platform, and the inferential enquiries were analyzed using the Statistical Package for Social Sciences (SPSS) version 21.

For the qualitative component, cool and warm analyses were done to create emerging themes based on the common patterns elicited from specific experiences [33]. Themes that emerged were validated through peer checking.

### 3.5. Research Ethics

To ensure the integrity of the study participants, the researchers secured a certification from an Institutional Ethics Review Board (IRB). The undertaking was designed to allow autonomy from the participants. Consent was secured from the participants and they were informed about the mechanics of the study. Participants who wished to leave the research were allowed without penalty and their personal information or any information taken from the participants was discarded from the study.

## 4. Results and Discussion

### 4.1. Participant Demographics

Respondent demographics (*n* = 107) (see Table 1) indicate that the participants were mostly female (80.4%) who were married (61.70%) and with 1–3 children (60.70%). They worked full time (91.60%) on private (72.90%) hospitals (68.20%). Their current position is directed towards staff nursing or clinical instruction (72.90%) receiving an average monthly salary of P15,000–P30,000 (69.20%).

Participants interviewed for the phenomenological component (*n* = 12) were returnees from a country in West Asia. They worked in the said country for 2–18 years ($\overset{-}{x}=11.8$ years) and have been staying in the Philippines for 4–15 years ($\overset{-}{x}=8.3$ years).

### 4.2. Association between the Length of Stay and Social Connectedness

Table 2 shows the agreement of the respondents in their social connectedness. Respondents in general had expressed positive social relationship with the people around them. It has to be noted that the scores were the respondents' latest perceived social connectedness.

Although the scores came consistent with all respondents ($\overset{-}{x}=4.75$, SD = 0.63, “agree”), it is interesting to note that items that returned slightly lower level of agreement from the respondents are those pertaining to communities (items 3, 6, and 15). It implies that under strictest scrutiny, Filipino nurse returnees might find less social connection from the community than from their family and peers. This may be explained by the innate orientation of Filipinos to families. It is known and has been reiterated by numerous researchers and experts alike that the Philippines is a family-oriented civilization and that the concept of self-esteem among Filipinos is linked to the bond between families and kinships [37–39]. The result may possibly be linked to the Filipinos' deep integration with families, deeper than its relationship with the community.

**Figure 1:**
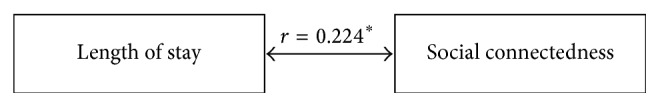
Resulted association between the quantitative variables.

Table 3 presents the resulted relationship between the participants' length of stay in the Philippines and their social connectedness. As shown in the table, the relationship between the two variables is of significance at 0.05 level with 107 participants (*r* = 0.224, *p* = 0.021). And based on Evans' [40] correlation strength interpretation, the association is weak. A graphical image for this association is also provided in Figure 1. This implies that the length of return is slightly correlated to the degree of social connectedness of the returnees. While the study result suggested a significant, but weak pattern on the factor of time and social connection, it is interesting to look deeper into its essence. The researchers turn their lenses on the multifactorial nature of social connectedness.

Lee and Robbins [41] associated social connectedness with trait anxiety, self-esteem, and social identity of a person. According to the research, trait anxiety and connectedness are inversely related; meaning low social connectedness results from a person with innate anxiousness. Social connectedness has also played major role in a person's self-esteem and identity. However, it is relevant to note that social connectedness, as suggested by the Lee and Robbins [41], only has relevance to self-esteem and identity when it is high, suggesting that high and low social connectedness may not even reside in the same continuum.

Besides trait anxiety, a person's personality is also linked to social connectedness. Studies suggest that extraversion or introversion affects how an individual relates to his or her society or the world. As a common trend observed by various researches [2, 4, 23, 24], people considered extroverted has been seen with high social connectedness [42], while the introverted people has been seen with the opposite [43].

Other researchers' take on social connectedness is rather technological than psychological in nature. McIntyre et al. [43] suggest that internet usage enhances the social connectedness, perhaps due to the extensive usage in social media sites among millennial and nonmillennial generation [44, 45] for seeking health information [45, 46], public opinions [47], search for current events [45], or simply socializing with communities across the globe [44]. Impulsive internet use, however, has been correlated with poor social connectedness [43].

While the former poised its gear towards technological factors for social connectedness, there are also studies focusing on political structures as concomitant to social cohesion. According to Rawatlal and Petersen [48], social relationship of communities is affected by certain variables such as political and managerial structures. Good policies and consistency in administrative management of the government promote comfort and satisfactory lifestyle. Lack of social resources, on the other hand, usually leads to social isolation among the people in the community [49]. Interpersonal and intrapersonal disciplines, or the innate social characteristic of the community and self, also play part in creating a sense of coherence of a person with his or her community [48].

Considering these associated elements, one may produce a very much viable model for social connectedness.

### 4.3. Qualitative Findings

**Figure 2:**
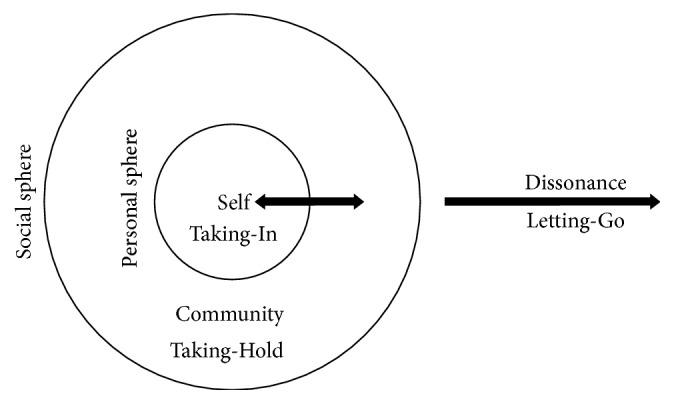
Social Connection System.

Upon cool and warm analysis of the participant transcription, a metaphor emerged to provide a visual analogy of the study findings. The Social Connection System (SCS; see Figure 2) was created to depict the essence of communication from the lens of Filipino nurse professionals as they permanently returned to their homeland.

The SCS consists of 2 overlapping circles and arrows with different endpoints. The inner sphere, or the personal sphere, represents the human self portrayed by the nursing professionals. They are inside the outer sphere, or the social sphere, which symbolizes the social community they are living with; they are, in the context of the SCS, the participants' family members, peers, coworkers, and their neighborhood.

In its purest form, the Social Connection System holds true that a person with high social connectedness stays within the social sphere, with the double headed arrow implicating that his or her experiences are normally geared towards him or herself or to his or her community. Experiences that are driven towards the person are intrapersonal in nature (Theme 1: Taking-In) and experiences driven towards the community are interpersonal in nature (Theme 2: Taking-Hold). A person with low social connectedness, however, loses his or her control of the gear and traverses from the social sphere to dissonance, a space characterized by detachment from the ecosystem of society (Theme 3: Letting-Go). The themes that surfaced with the eidetic imagery are summarized in Table 4.

The essence of communication among the participants is characterized by both bittersweet and distressful encounters with themselves and the people that surround them. Fundamentally, the life of a professional nurse is never an easy life, the moment he or she steps away from his or her homeland to practice the profession. When one returns home, he or she receives warm welcomes and sweet reunions from family and friends, but returns are not always a happy ending [50, 51]. The themes that materialized in the study mark this assertion.

#### 4.3.1. Theme 1: Taking-In

Taking-In is characterized by introverted connections with oneself. These are the experiences of Filipino nurse migrants that are directed towards their own feelings. Basically, the experiences were taken in and determined the person's relationship with themselves. In the context of connectedness, this relationship with oneself is positive in general.

Return migrants often reap what they seeded after going back to their homeland. For years, they worked in a different country, with different socioeconomic levels, mostly higher than their homeland [52]. Often than not, they acquire critical skills that were not learned before, polish the skills, and master them. As they return, they possess the conviction in their profession. As some participants expressed, “*…I felt more confident upon returning.”*,* “…I had this confidence that there will always be away to resolve problems and face them.”*. Confidence overflows from them that they lift their profession up in the company. Some verbalized,* “I can say that I am stronger. Like in my line of work, I am able to face doctors, patients and co-workers and explain what should be done.”* and* “I wanted to make a difference in my profession. I tried to implement the policies I observed from abroad. I guess it worked and they saw how well I was able to manage the nursing service.”*

Their confidence also transpired as they socially connected with people of different status and nationalities. Their interactions to variety of personalities, more unique than their country of origin, taught them new ways of communicating and handling people. Some even underwent vast changes in their personality, from being a self-reserved person to a socially confident professional. Two particularly described,* “With my experience of working with different nationalities, I felt I can handle similar situations in the work place [as I return home].”*, and* “From a shy, no-talk woman raised by nuns and two aunties, I became an expressive person. I guess I got my confidence from working abroad and mingling with different people.”*

It is natural that migrants learn critical skills and experiences which are useful to them. These skills refer to a broad area including specialized skills, that is, skills that are specific for nursing and clinical services, and social skills, communication skills learned from interaction with people of different nationalities, with different characteristics and personalities. Most of these skills are acquired through job training [12].

Another benefit one can get from working abroad is gaining financial stability. It is always known that nursing jobs in developed countries offer more satisfying salary than ones from developing countries and that nursing professionals often work in these countries to earn more [14]. Many participants suggest personal satisfaction, as indicated* “Benefits of course pertains to financial aspects. I was able to provide for them. We were able to buy our own house and save some.”, “Professionally, I have settled down already. I was able to finish my graduate studies so I can say that I am fulfilled.”*, and* “I do not have any draw backs on migration.”*

It is reflected upon the experiences of nurse returnees the benefits they reaped after sowing hard work away from their comfort zone. And since all the participants in this study are females who are married, these benefits, obviously, are accordingly shared by nurse migrants to their loved ones, their family and friends, as they settled in their country of origin for good.

Also, in the light of exploring on the experiences of the participants, the researcher noted that experiences drawn towards self are inevitable. When one explores on the social connectedness of a person to another, personal affectations often show. This is evident on the semiformal interview done by the researchers to the Filipino nurse returnees, as they share their experiences of their return.

#### 4.3.2. Theme 2: Taking-Hold

Taking-Hold is characterized by extroverted connections of the participants to the people around them. In this theme, the focus turns from the personal experiences of the nurse migrants towards their society and the personalities around them. This includes their family, mostly their children, their friends, colleagues, and their neighborhood.

The context of taking hold is also positive to neutral in nature. This describes a picture of nurse returnees that had satisfactory experiences with people as they returned to their country. In a broader sense, it involves keeping in touch with friends, catching up to lost times with families, and servicing community.

When a person goes abroad, he or she diminishes his or her role in the place he or she left. It may be his or her role as a parent, as a child, as a spouse, or as a friend. Nurse migrants in the study reached out to their loved ones as they returned home. They need to recuperate for the lost times when they worked outside the country.

Some of them who lost their roles as a parent or a spouse came back and assumed their roles. This is their way of reconnecting with their loved ones and improving social coherence between them. One parent describes how she worked hard for it,* “I went out of my way to reintegrate myself back to my family's arm. I showed them that they are dear to me in anyway I can. I cooked for them, went to school activities, attended to my husband – the works. Economically, I assisted my husband in our business….”*

*“I also had to reintegrate myself back to the lives of my husband and children on their activities, ways, likes and dislikes.”*

Another parent proudly told her story, as she said,* “As I went home, I concentrated with caring for our only son. My husband wanted me to guide our son on his education because at that time, he was doing poorly at school. I concentrated on being his personal tutor at home and we were so happy that he made it to back to the top of his class.”*

The most valuable price for this is the acceptance they get from their family, as one articulated,* “…acceptance has always been there from my family… I re-established myself back with my family.”*, while another said* “It took some time but after a while I was able to adjust.”*

Besides recuperating with their family, professional nurse returnees also find time getting in touch with their friends. And in line with this, the most precious experience one can get is the feeling of acceptance from the community they are in. One verbalized,* “They have become my friends as well. They welcomed me with open arms.”*

Some of the returnees also reached out to their community through providing them service. Many of them returned and were able to continue working. This is one returnee who conveyed her experience as a social worker in the community:* “In the community, I participated in more activities in the church. I was able to handle projects and programs related to health. I felt that in my own small way I was able to help.”*

One participant got a job as a clinical instructor.* “I felt appreciated when I worked as a clinical instructor particularly when I shared my experiences abroad to my students. They were very eager to learn from me. I was fortunate to be hired right away.”*

Another participant has landed in the clinical setting.* “I was answered by the opportunity given by our church when they hired me to head our hospital.”*

Companies and institutions often look for skills and years of experiences when searching for employees. When these nurses returned home, they brought critical skills [12] in patient care and management with them, giving them higher chances of being trusted by employers.

#### 4.3.3. Theme 3: Letting-Go

Letting-Go happens when the person loses connectedness to the society. Generally negative in its sense, this disconnection may be initiated by society or the person himself. Nevertheless, the result is inharmoniousness between the person and her family, or friends or the neighborhood.

One of the experiences that the returnees expressed is the feeling of detachment from the community they are living with. It is characterized by discomfort with the people outside their houses. One respondent verbalized “I was not in my comfort zone,” when asked about her community when she returned.

Filipinos have this fond characteristic of asking for presents from OFWs when they return [51]. While this can be a source of friendly interaction with neighbors, some returnees may perceive it as shameful attitude of the neighbors. One Filipino nurse returnee verbalized “Sometimes, I felt uncomfortable relating with my neighbors – that they might ask something from me.”

The lack of social connection towards their neighbor can probably be connected to the family orientation of Filipinos. Filipinos are naturally family centered and may put more effort in connecting to their family members than the people outside their house [37–39]. On the contrary, Filipinos are also considered sociable by many scholars [53]. It makes it interesting as to how Filipino nurse returnees tend to lose their social connectedness when they return.

This may simply be explained by differences in personalities and social skills of persons. Some suggest that while Filipinos are sociable in nature, they are also sensitive people and that conflicts are normally occurring when differences in needs, values, and personalities come together [53].

Another experience of the study participants is their hardship when relating to the family they left for a long time. Children who were left by their mothers when they were young find it difficult to understand the situation that their mothers had to go through. This results in miscommunication between the child and the parent. One participant shared her encounter with her child:

*“I was affected by the reaction of my youngest child. I cried because no matter what I do, he seems to be very angry at me. When I tried to ask why, the answer was ‘Because you left me.' I was not expecting that, then I realized that they have not yet understood that I left to work abroad for their welfare.”*

It is even hard when the child found the care of a primary care giver from a different person. This gives the participants a feeling of indifference from the family members, as one participant expressed,* “Drawback could be the distance I felt from my children…”*. Some participants shared their experience as their children found comfort from the hands of a person other than them:

*“The only drawback I can share is the lack of closeness between me and my children. They seem to be more comfortable with their father. They usually reason that they were hesitant in asking me for help because they're uncomfortable.”*

*“The hardest part of my return was when my children cried whenever I take care of them like giving a bath. They like our maid better. They did not even want to sleep in our room because they do not know me.”*

Children need a primary caregiver as they grow up. Normally, the child perceives the primary caregiver role from his or her mother. However, when this is impossible to happen, for instance, the mother is working abroad, the child will seek a primary caregiver from the hands of another. It can be gotten from the father, who usually is left with the child, or the house helper [54].

For years, OFWs worked away from their significant others, and flying back home has always been the most exciting thing they think about. As they return they expect to see the faces of the people they left, only to find out that it is not anymore the way it was before. In this instance, the returnees have become a stranger in their own country [51].

## 5. Conclusion and Recommendations

A mixed method was exploited in this cross-sectional research in order to expound on social connectedness among Filipino nurse professionals who returned to the country permanently. Nested concurrent for mixed method is designed to allow researchers to simultaneously conduct both the quantitative and qualitative phases of the research. The quantitative phase utilized a descriptive correlational design to determine the association between the respondents' length of stay after returning to the country and their social connectedness with the community. The qualitative phase applied interpretative phenomenology to depict the essence of communication from the lens of the participants. The interpretative type also negates the necessity of bracketing, allowing the assigned female interviewer who is married to get key informants with the same characteristics as hers.

From the 107 participants, the respondents generally expressed positive social relationship with people. It implies that the respondents are currently in harmonious relationship with their family, friends, and the community where they live. Their social coherence with the community, however, received the lowest agreement. This is linked then by the researchers to the natural affinity of the Filipinos to their family.

The difference between the scores in social connectedness with community against family and peers is not determined in this study. Therefore, the researchers recommend to further examine this matter.

The Pearson-*r* returned significant but weak association between the time and social connectedness of the nurse returnees. The pattern presented as a result seems inconclusive because of the responses of the participants which lean towards strong agreement (agree and strongly agree). The researchers then turned their focus to other factors that were claimed to have a relationship with social coherence of a person to another. The researchers strongly suggest more participants with well-distributed responses to print a better pattern of time and social connectedness. Focusing on the multifactorial phenomenon of social connectedness of people may also be beneficial.

Due to its limitations, the results of the study may not be representative to the whole population of nurse migrants. Generalizability of the study results can be further established through replication with further recruitment of participants spanning to wider locale in the country in consideration by scholars in the field.

Another limitation on the quantitative phase rests upon the usage of snowball sampling due to unavailability of official data for returned nurse migrants. Future researchers may look back for updates so they can utilize a better technique in electing respondents.

Upon the qualitative valuation of the study, three themes have emerged. Taking-In is the intrapersonal experiences of the Filipino nurses who returned to the country permanently. It is described by sense of worth, feeling of belongingness, security, and overall satisfaction among the participants. Taking-Hold is the extroverted connection between the participants and their family and neighbor. It entails their stories of reaching out to their loved ones and their community. Letting-Go, on the other hand, is depicted by their negative experiences with their family and the community.

In line with the qualitative findings, the researchers intend to emphasize on constant communication of nurse OFWs with their family and friends as they work abroad in order to sustain positive relationship with them.

It should be noted, however, that the themes that emerged are coming specifically from Filipino female nurses who are married. Exploring the perspective of other Filipino nurses, such as male nurses or nurses who are single, is then recommended by the researchers. It is therefore advised to consider expanding the team of interviewers, specifically when the researchers follow a methodological principle wherein the participants with similar characteristics as the interviewers will be employed.

The Social Connection System (SCS) is the visual analogy that was created in the study that embodies the structure of connectedness of a person with its society. A person that is inside the social sphere is considered having high social connectedness. A person with low connection drives themselves outside the sphere into dissonance, a space of disconnection from the community.

## Figures and Tables

**Table 1:** Demographic profile of the respondents (*n* = 107).

	*f*	%
Gender		
Female	86	80.4
Male	20	18.7
Marital status		
Married	66	61.7
Separated	8	7.5
Single	31	29
Widow	2	1.9
Number of children		
0	36	33.6
1–3	65	60.7
4–7	6	5.6
Current employment		
Full time	98	91.6
Part time	8	7.5
No response	1	0.9
Type of company		
Private	78	72.9
Public	29	27.1
Area of practice		
Hospital	73	68.2
School	34	31.8
Current position		
AVP NDIV	1	0.9
Chief nurse	2	1.9
Dean	3	2.8
Manager	20	18.7
Staff	78	72.9
Training officer	2	1.9
Salary		
15,000–30,000	74	69.2
31,000–45,000	22	20.6
46,000–60,000	6	5.6
61,000 & above	5	4.7

Gathered in June to July 2015 from various areas in Metro Manila and Bulacan, Philippines.

**Table 2:** Mean scores of respondents' social connectedness (*n* = 107).

	Mean	SD	Interpretation
(1) I feel at ease in the presence of outsiders.	4.61	1.09	Agree
(2) I am accustomed with the community where I work and live with.	4.99	0.69	Agree
(3) I have not developed personal attachment outside my family since I returned from abroad.^*∗*^	4.47	1.19	Slightly Agree
(4) I have readjusted well upon return.	4.89	0.94	Agree
(5) I feel a sense of belongingness to those around me.	4.85	0.94	Agree
(6) I feel detached from the community where I live and work.^*∗*^	4.49	1.21	Slightly Agree
(7) I have not developed close attachment even with my own family.^*∗*^	4.93	1.03	Agree
(8) I view people as pleasant and open-minded.	4.79	1.03	Agree
(9) I feel like a stranger (*n* = 106)^*∗*^.	4.87	1.05	Agree
(10) I feel appreciated by those around me.	4.70	0.91	Agree
(11) I feel isolated from those around me.^*∗*^	4.81	1.11	Agree
(12) I am able to connect with my peers.	4.88	0.93	Agree
(13) I do not feel that I am accepted by my peers.^*∗*^	4.72	1.12	Agree
(14) I am much concerned with the welfare of others.	4.75	0.95	Agree
(15) I find myself less attached with my community.^*∗*^	4.42	1.21	Slightly Agree
(16) I am able to relate with other people.	4.93	0.92	Agree
(17) I view myself as an outsider since my return.^*∗*^	4.73	1.11	Agree
(18) I do not feel an attachment to others.^*∗*^	4.61	1.19	Agree
(19) I consider my friends as family.	4.96	0.93	Agree
(20) I sense a lack of connectedness with others.^*∗*^	4.65	1.10	Agree
Overall	4.75	0.63	Agree

Strongly Disagree 1–1.49; Slightly Disagree 1.5–2.49; Disagree 2.5–3.49; Slightly Agree 3.5–4.49; Agree 4.5–5.49; and Strongly Agree 5.5–6.0.

^*∗*^Reversed scoring.

**Table 3:** Relationship between length of stay and social connectedness (*n* = 107).

	*r*	*p* value	Association
Length of return *↔* social connectedness	0.224^*∗*^	0.021	Weak^*∗∗*^

^*∗*^Correlation is significant at the 0.05 level.

^*∗∗*^Based on correlation interpretation by Evans [40].

**Table 4:** Emerged themes and description.

Themes	Description
Taking-In	Intrapersonal connectedness characterized by sense of worth, gradual feeling of belongingness, fulfillment of skills, financial security, and overall satisfaction

Taking-Hold	Connection between the person and others portrayed by sense of coherence with peers, acceptance from the family, and service to community

Letting-Go	Expected events that did not occur, resulting in discordance between family members and detachment from the community
